# Case Report: An EGFR-Targeted 4-1BB-agonistic Trimerbody Does Not Induce Hepatotoxicity in Transgenic Mice With Liver Expression of Human EGFR

**DOI:** 10.3389/fimmu.2020.614363

**Published:** 2021-01-07

**Authors:** Marta Compte, Seandean L. Harwood, Jorge Martínez-Torrecuadrada, Gema Perez-Chacon, Patricia González-García, Antonio Tapia-Galisteo, Paul M. P. Van Bergen en Henegouwen, Aránzazu Sánchez, Isabel Fabregat, Laura Sanz, Juan M. Zapata, Luis Alvarez-Vallina

**Affiliations:** ^1^ Department of Antibody Engineering, Leadartis SL, Madrid, Spain; ^2^ Department of Molecular Biology, Aarhus University, Aarhus, Denmark; ^3^ Spanish National Cancer Research Center (CNIO), Madrid, Spain; ^4^ Instituto de Investigaciones Biomédicas Alberto Sols (IIBm), CSIC-UAM, Madrid, Spain; ^5^ Instituto de Investigación Sanitaria La Paz (IdiPaz), Madrid, Spain; ^6^ Molecular Immunology Unit, Hospital Universitario Puerta de Hierro Majadahonda, Madrid, Spain; ^7^ Division of Cell Biology, Department of Biology, Science Faculty, Utrecht University, Utrecht, Netherlands; ^8^ Department of Biochemistry and Molecular Biology, Faculty of Pharmacy, Complutense University of Madrid (UCM), Health Research Institute of the Hospital Clínico San Carlos (IdISSC), Madrid, Spain; ^9^ Oncobell Program, Bellvitge Biomedical Research Institute (IDIBELL), CIBEREHD and University of Barcelona, L’Hospitalet de Llobregat, Barcelona, Spain; ^10^ Cancer Immunotherapy Unit (UNICA), Department of Immunology, Hospital Universitario 12 de Octubre, Madrid, Spain; ^11^ Immuno-Oncology and Immunotherapy Group, Hospital 12 de Octubre Biomedical Research Institute (imas12), Madrid, Spain

**Keywords:** cancer immunotherapy, immunostimulatory antibodies, 4-1BB agonists, hepatotoxicity, trimerbodies, EGFR, EGFR-targeted 4-1BB agonists

## Abstract

Agonistic monoclonal antibodies (mAbs) targeting the co-stimulatory receptor 4-1BB are among the most effective immunotherapeutic agents across pre-clinical cancer models. However, clinical development of full-length 4-1BB agonistic mAbs, has been hampered by dose-limiting liver toxicity. We have previously developed an EGFR-targeted 4-1BB-agonistic trimerbody (1D8^N/C^EGa1) that induces potent anti-tumor immunity without systemic toxicity, in immunocompetent mice bearing murine colorectal carcinoma cells expressing human EGFR. Here, we study the impact of human EGFR expression on mouse liver in the toxicity profile of 1D8^N/C^EGa1. Systemic administration of IgG-based anti-4-1BB agonist resulted in nonspecific immune stimulation and hepatotoxicity in a liver-specific human EGFR-transgenic immunocompetent mouse, whereas in 1D8^N/C^EGa1-treated mice no such immune-related adverse effects were observed. Collectively, these data support the role of FcγR interactions in the major off-tumor toxicities associated with IgG-based 4-1BB agonists and further validate the safety profile of EGFR-targeted Fc-less 4-1BB-agonistic trimerbodies in systemic cancer immunotherapy protocols.

## Introduction

The success of immune checkpoint blockade using PD-1/PD-L1 and/or CTLA-4 inhibitors has validated the concept of immunomodulating monoclonal antibodies (mAbs) as a powerful therapeutic strategy, but responses are still limited to a minor fraction of cancer patients (1). Immune cell stimulation by agonistic mAbs acting on co-stimulatory receptors, such as CD40, OX40, and 4-1BB, is a particularly interesting approach, as these receptors are mainly expressed on T cells upon activation (2, 3). 4-1BB (CD137, TNFRSF9) is a member of the tumor necrosis factor receptor superfamily (TNFRSF) that is transiently expressed following activation through the T cell receptor (TCR) (4). To date, a unique ligand for 4-1BB has been identified, 4-1BBL (TNFSF9), which is expressed on the surface of antigen-presenting cells (5). 4-1BBL trimerization leads to 4-1BB receptor clustering and TRAFs-mediated activation of NF-κB and MAPK intracellular signaling cascades leading to enhanced T cell proliferation and survival (6).

However, off-tumor toxicities have been the major impediment to the clinical development of first-generation IgG-based 4-1BB agonistic mAbs. The fully human IgG_4_ urelumab caused dose-dependent liver toxicity, including two fatalities (7, 8). Additional studies have shown that dose reduction ameliorated liver toxicity, but also resulted in limited clinical activity (8). The fully human IgG_2_ utomilumab displayed a better safety profile but is a relatively less potent 4-1BB agonist (9). Therefore, new strategies are being developed to preserve the anti-tumor effect avoiding off-tumor toxicities associated with FcγR interactions (10–12). These approaches aim to confine 4-1BB co-stimulation to the tumor microenvironment.

We have recently described a novel EGFR-targeted Fc-less 4-1BB agonistic trimerbody (1D8^N/C^EGa1), which is a potent costimulator *in vitro* and exhibits enhanced tumor penetration and powerful anti-tumor activity in immunocompetent mice bearing gene-modified CT26 colorectal carcinoma cells expressing human EGFR (10). In this model, the anti-tumor effect of the bispecific trimerbody was dependent on human EGFR expression (13), but the potential toxicity profile was dictated by the endogenous mouse EGFR. In this context, the 1D8^N/C^EGa1 trimerbody did not induce the systemic cytokine production and hepatotoxicity associated with IgG-based 4-1BB agonists (10). To further investigate this aspect and given that the anti-EGFR EGa1 V_HH_ single-domain antibody was isolated from a phage-displayed llama V_HH_ library immunized with EGFR-positive human cells (14, 15), we studied here the impact of human EGFR expression on the liver in the 1D8^N/C^EGa1 toxicity profile in a liver-specific huEGFR-transgenic immunocompetent mouse (16). In this model, systemic administration of IgG-based anti-4-1BB agonist resulted in nonspecific immune stimulation and liver toxicity, whereas treatment with the EGFR-targeted 4-1BB-agonistic trimerbody lacked these immune-related side effects.

## Methods

### Mice

C57BL/6 wild-type (WT) female mice and transgenic Alb-Δ^654–1186^huEGFR (ΔEGFR-tg) (16) littermates were housed in the animal facility of the Instituto de Investigaciones Biomédicas “Alberto Sols” (IIBm) (CSIC-UAM, Madrid, Spain). Animals were kept in controlled conditions of temperature (21 ± 1°C), humidity (50 ± 5%), and 12 hours light/dark cycles. Manipulation was performed in laminar flow hood, when necessary, and sterilized water and food were available ad libitum All animal procedures conformed to European Union Directive 86/609/EEC and Recommendation 2007/526/EC, enforced in Spanish law under RD 1201/2005. Animal protocols were approved by the Animal Experimentation Ethics Committee of the IIBm, and the Animal Welfare Division of the Environmental Affairs Council of the Government of Madrid (66/14, 118/19).

### Cells and Culture Conditions

HEK293 (CRL-1573) cells were obtained from the American Type Culture Collection and mouse CT26 cells (CRL-2638) expressing human EGFR (CT26^huEGFR^) or infected with the empty vector retrovirus (CT26^mock^) were provided by Dr M. Rescigno (European Institute of Oncology, Milan) (13). The cells were grown in complete Dulbecco’s modified Eagle’s medium (DMEM) (Lonza) supplemented with 2 mM L-glutamine, 10% (vol/vol) heat-inactivated Fetal Calf Serum (FCS), and antibiotics (100 units/mL penicillin, 100 mg/mL streptomycin) (all from Life Technologies) referred as to DMEM complete medium (DCM), unless otherwise stated. The cell lines were routinely screened for mycoplasma contamination by PCR (Stratagene).

### Hepatocyte Isolation and Culture

Hepatocytes were isolated as previously described following the two-step collagenase perfusion technique followed by isodensity purification in a Percoll gradient (17). Briefly, livers from 3 months-old mice were perfused with Hanks´ balanced salt solution supplemented with 10 mM Hepes and 0.2 mM EGTA for 5 min, followed by a perfusion (10–15 min) with William´s E medium containing 10mM Hepes and 0.03% collagenase I (Worthington). Livers were further minced, and viable hepatocytes were selected by centrifugation in Percoll and seeded in collagen I-coated plates (5 µg/sq cm) at a density of 28 x 10^3/^cm^2^ in Dulbecco´s modified Eagle´s medium/F-12 (1:1) supplemented with 10% serum.

### Expression and Purification of Recombinant Antibodies

The 1D8^N/C^EGa1 trimerbody was produced in stably transfected HEK293 cells (10) cultured in complete DMEM with 500 μg/mL G418 (all from Life Technologies), and conditioned medium purified using the (Twin-)*Strep*-tag purification system (IBA Lifesciences) connected to an ÄKTA Prime plus system (GE Healthcare). The purified antibody was dialyzed overnight at 4 °C against PBS + 150 mM NaCl (pH 7.0), analyzed by SDS-PAGE under reducing conditions and stored at 4°C. Purified antibody was tested for endotoxin levels by Pierce´s limulus amebocyte lysate (LAL) chromogenic endotoxin quantitation kit, following the manufacturer’s specifications (Thermo Fisher Scientific). Endotoxin levels of purified antibody stocks were lower than 0.25 EU/ml as determined by LAL test. Purified anti-mouse 4-1BB IgG (clone 3H3) was purchased from (cat#BE0239, BioXCell).

### ELISA

Purified mouse 4-1BB:hFc chimera (mo4-1BB), mouse EGFR:hFc (moEGFR) and human EGFR:hFc chimera (huEGFR) (all from R&D Systems) were immobilized at 3 µg/ml on Maxisorp ELISA plates (NUNC Brand Products) overnight at 4 °C. After washing and blocking with 200 µl PBS 5% BSA (Merck Life Science), 100 µl of purified 3H3 IgG or 1D8^N/C^EGa1 trimerbody were added and incubated for 1 hour at room temperature. The wells were washed for three times with PBS 0.05% Tween-20, and 100 µl of anti-FLAG mAb (clone M2; mIgG_1_; cat#F1804, Merck Life Science) were added for 1 hour incubation at room temperature. The plate was washed as above and 100 µl of HRP-conjugated goat anti-rat IgG or HRP-conjugated goat anti-mouse IgG (both from Merck Life Science) were added to wells previously incubated with 3H3 IgG or 1D8^N/C^EGa1 trimerbody, respectively. Afterwards, the plate was washed and developed using OPD (Merck Life Science).

### Biolayer Interferometry

All biolayer interferometry was performed on an Octet RED96 (Fortebio). To investigate the binding of 1D8^N/C^EGa1 to hu-EGFR or moEGFR, 30 nM of huEGFR or moEGFR in fusion with a human Fc region were immobilized onto AHC biosensors (Fortebio) coated with anti-human Fc antibodies for 20 min, in 20 mM HEPES, 150 mM NaCl pH 7.4 buffer (HBS). Then, biosensors were moved into 20 nM 1D8^N/C^EGa1 in HBS and association was measured for 20 min followed by one hour of dissociation in HBS. To investigate the binding of hu-EGFR or moEGFR in solution to immobilized 1D8^N/C^EGa1, biosensors coated with mo4-1BB in fusion with a human Fc region were prepared using amine reactive chemistry. Briefly, AR2G biosensors (Fortebio) were activated with s-NHS/EDC, coated with 2 µg mouse 4-1BB per biosensor at pH 6 for 20 min, and quenched with ethanolamine. Then, 10 nM of 1D8^N/C^EGa1 in HBS was immobilized onto the biosensors for 30 min. Human or moEGFR (50 nM in HBS) was then introduced and allowed to associate for 20 min and dissociate for one hour. In both experiments, a reference biosensor coated and immobilized with the same ligands, but not receiving the experimental analyte proteins, was subtracted from the other sensorgrams prior to data analysis. Data were fit to 1:1 binding models using the Octet Data Analysis software (Fortebio). In the case of moEGFR’s binding to immobilized 1D8^N/C^EGa1, fitting included only its initial association phase, due to its biphasic binding.

### Flow Cytometry

The cell surface expression of EGFR was analyzed on freshly-isolated liver cells from C57BL/6 WT and EGFR-tg mice, and on CT26^mock^ and CT26^huEGFR^ cells after incubation for 30 min with the human EGFR-specific chimeric mouse/human IgG_1_ cetuximab (Merck KGaA), or the purified 1D8^N/C^EGa1 trimerbody. After washing, cells were treated with appropriate dilutions of phycoerytrin (PE)-conjugated goat anti-human IgG F(ab′)_2_ (Fc specific; cat#109-116-097, Jackson Immuno Research), or anti-FLAG mAb (clone M2), and then with PE-conjugated goat anti-mouse IgG F(ab’)_2_ antibody (cat#115-116-072, Jackson Immuno Research). Samples were analyzed with a MACSQuant Analyzer 10 flow cytometer (MiltenyiBioteh). A minimum of 20,000 events were acquired for each sample and data were evaluated using FCS Express V3 software (De Novo Software).

### Toxicity Studies

Eight weeks old C57BL/6 wild-type and ΔEGFR-tg littermates received a weekly i.p. dose of 3H3 IgG or 1D8^N/C^EGa1 (6 mg/kg) for 3 weeks. Mice were anesthetized and bled on days 0, 7, 14, and 21. To obtain mouse serum, blood was incubated in BD microtainer SST tubes (BD Biosciences), followed by centrifugation. Serum was stored at −20 °C until use. Serum levels of alanine aminotransferase (ALT) were determined at day 14 using Reflotron GPT/ALT strips and the Reflotron plus analyzer (Roche Diagnostics). One week after the last dose of antibodies, mice were euthanized and the liver and spleens, were surgically removed, weighted, and fixed in 10% paraformaldehyde for 48 h. Then fixed tissues were washed and embedded in paraffin. Tissue sections (5 µm) were stained with hematoxylin and eosin. Lymphocyte infiltration in the liver was quantified using the ImageJ software.

### Histological Studies

Tissue samples were fixed in 10% neutral buffered formalin (4% formaldehyde in solution), paraffin-embedded and cut at 3 μm, mounted in superfrost^®^ plus slides and dried overnight. For different staining methods, slides were deparaffinized in xylene and re-hydrated through a series of graded ethanol until water. Consecutive sections for several immunohistochemistry reactions were perform in an automated immunostaining platform (Ventana Discovery XT, Roche; AS Link, Dako, Agilent). Antigen retrieval was first performed with the appropriate pH buffer, (CC1m, Ventana, Roche; Low pH buffer, Dako, Agilent) and endogenous peroxidase was blocked (peroxide hydrogen at 3%). Then, slides were incubated with the appropriate primary antibody as detailed: rabbit monoclonal anti-EGFR (mouse preferred) (D1P9C, 1/600, Cell Signaling, #71655) and mouse monoclonal anti-huEGFR (EGFR.113, 1/10, Leica, NCL-EGFR). After the primary antibody, slides were incubated with the corresponding visualization systems (OmniMap anti-Rabbit, Ventana, Roche; EnVisionFLEX+Mouse Linker, Dako, Agilent) conjugated with horseradish peroxidase. Immunohistochemical reaction was developed using 3, 30-diaminobenzidine tetrahydrochloride (ChromoMap DAB, Ventana, Roche; FLEX DAB, Dako, Agilent) and nuclei were counterstained with Carazzi’s hematoxylin. Finally, the slides were dehydrated, cleared and mounted with a permanent mounting medium for microscopic evaluation. Positive control sections known to be primary antibody positive were included for each staining run. Whole slides were acquired with a slide scanner (AxioScan Z1, Zeiss).

### Statistical Analysis

Statistical analysis was performed using GraphPad Prism Software version 6.0. Data is presented as mean ± SD. Significant differences (*P* value) were discriminated by applying a two-tailed, unpaired Student’s *t* test assuming a normal distribution. *P* values are indicated in the corresponding figures for each experiment.

## Results and Discussion

### The 1D8^N/C^EGa1 Trimerbody Binds to Human EGFR With a Higher Affinity Than to Mouse EGFR

**Figure 1:**
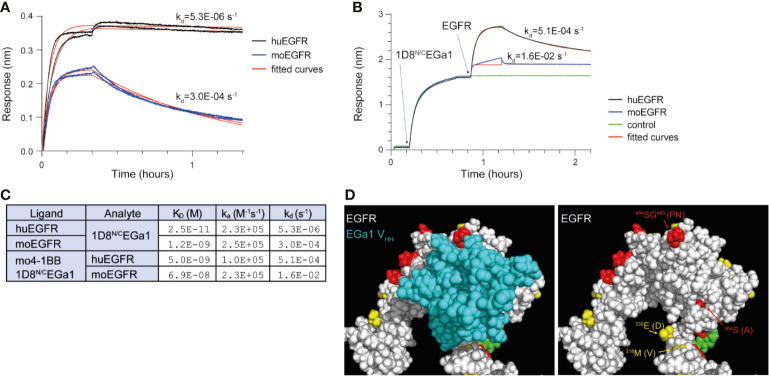
Biolayer interferometry investigating the binding of 1D8^N/C^EGa1 to human and mouse EGFR. **(A)** Human EGFR (huEGFR) and mouse EGFR (moEGFR), both in fusion with a human Fc region, were immobilized onto biosensors coated with anti-human Fc antibodies prior to the experiment. 20 nM of 1D8^N/C^EGa1 associated with the biosensors for 20 min, followed by one hour of dissociation. Duplicate biosensors are shown, along with theoretical binding curves for the kinetic rate constants obtained by fitting. **(B)** 10 nM of 1D8^N/C^EGa1 was immobilized to biosensors coated with mouse 4-1BB for 30 min, after which 50 nM of human or mouse EGFR (both in fusion with a human Fc region) associated for 20 min and dissociated for one hour. Theoretical binding curves are shown; note that fitting to mouse EGFR’s association step was limited to the first binding phase, due to its heterogeneous binding. **(C)** Kinetic rate constants and dissociation constants were obtained by fitting of the experimental binding data from panels **(A**, **B)** to 1:1 binding models. In both experiments, 1D8^N/C^EGa1 dissociates more rapidly from moEGFR than from huEGFR. **(D)** The crystal structure of the EGa1 V_HH_ bound to human EGFR (PDB 4KRO), shown with and without the EGa1 V_HH_. Residues of EGFR that are conserved between huEGFR and moEGFR are colored white, while similar residues are yellow, dissimilar residues are red, and glycans are green. Differing residues in proximity to EGa1 are labeled with the murine residue in parenthesis.

The EGa1 is a well characterized EGFR-specific V_HH_ that was generated from a phage-displayed llama V_HH_ library after immunizing and screening with EGFR-positive human cells (14, 18). Binding studies using biolayer interferometry were used to compare the binding of EGa1V_HH_ to human and mouse EGFR when integrated in a multichain bispecific anti-4-1BB x anti-EGFR trimerbody format (**Figure S1**) (10). These interactions were investigated in two orientations, either with biosensor-immobilized EGFR and 1D8^N/C^EGa1 in solution (**Figure 1A**), or immobilized 1D8^N/C^EGa1 and EGFR in solution (**Figure 1B**). In both orientations, the interaction between 1D8^N/C^EGa1 and human EGFR (huEGFR) dissociated much more slowly than the interaction between 1D8^N/C^EGa1 and mouse EGFR (moEGFR); for biosensor-immobilized EGFR and 1D8^N/C^EGa1 in solution, the interaction half-lives were ~36 hours and ~40 min for human and mouse EGFR, respectively, while for the reversed orientation, the half-lives were ~20 and ~1 min (**Figure 1C**). The difference in measured dissociation rates in the two orientations probably reflects differences in avidity due to trivalent binding by 1D8^N/C^EGa1 and bivalent binding by EGFR (fused to a human Fc region). A comparison of the primary sequence of huEGFR and moEGFR showed that EGa1’s epitope, as seen in the 4KRO crystal structure (19), is mostly conserved, with four differing residues around the periphery of the epitope (**Figure 1D**). This is consistent with the lower affinity of EGa1 for moEGFR determined by these binding studies.

### The 1D8^N/C^EGa1 Trimerbody Shows Negligible Toxicity in Immunocompetent Transgenic Mice Expressing Human EGFR in the Liver

**Figure 2:**
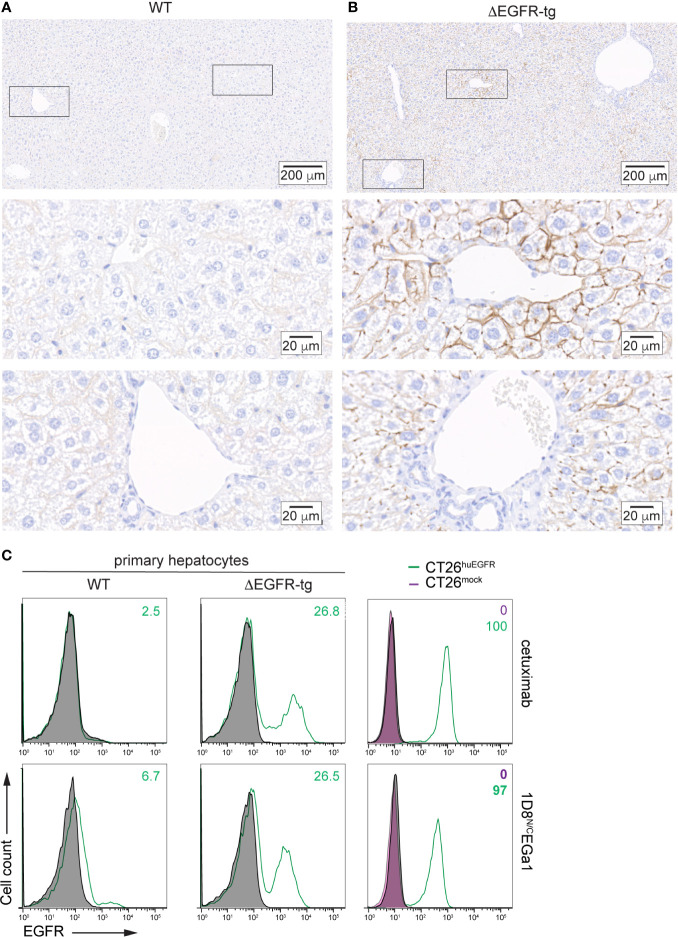
Analysis of human EGFR expression by IHC in liver sections from **(A)** WT C57BL/6 and **(B)** ΔEGFR-tg mice. Representative micrographs of liver sections from the indicated mice showing human EGFR staining in brown. Nuclei are counterstained with hematoxylin. Scale bars, 200 µm (upper images); and 20 µm (middle and lower images). Upper panels show panoramic views of the corresponding mouse livers, and middle and lower panels are higher magnification images of centrilobular (zone 3) and periportal areas (zone 1), respectively, corresponding to zones indicated by black boxes in the upper images. **(C)** Flow cytometry analysis of EGFR expression in primary hepatocytes freshly isolated from WT C57BL/6 and ΔEGFR-tg mice (right panels) or CT26^mock^ and CT26^huEGFR^ cells (left panels). Cells incubated with isotype control antibodies are shown as grey-filled histogram. Fluorescence intensity (abscissa) is plotted against relative cell number (ordinate). The numbers indicate the percentage of EGFR-positive cells.

Transgenic Alb-Δ^654–1186^EGFR mice (from now abbreviated as ΔEGFR-tg) are immunocompetent animals expressing an hepatocyte-specific truncated form of the human EGFR that lacks the intracellular catalytic domain (amino acids 654–1186) (16). Liver paraffin sections from wild-type C57BL/6 (WT) and ΔEGFR-tg mice were stained with moEGFR-specific and huEGFR-specific mAbs. In WT and ΔEGFR-tg mice, hepatocytes showed moEGFR expression on the entire surface of the cytoplasmic membrane with a strong and uniform intensity in most of the liver lobules (**Figure S2**). In ΔEGFR-tg mice, hepatocytes showed a partial and segmental expression of huEGFR in the cytoplasmic membrane with a strong intensity distributed in segments or areas of different sizes, sometimes exhibiting a punctiform pattern, especially in centrilobular hepatocytes (**Figure 2B**). Periportal and midzonal hepatocytes displayed also huEGFR expression albeit at a lesser extent (**Figure 2B**). No expression of huEGFR was detected in WT mice (**Figure 2A**). These findings were further confirmed by flow cytometry, where it was also found that about 25% of freshly isolated primary hepatocytes from ΔEGFR-tg mice expressed significant levels of cell surface huEGFR (**Figure 2C**), and that the 1D8^N/C^EGa1 trimerbody is more efficient in recognizing hepatocytes isolated from ΔEGFR-tg mice than those from WT mice (**Figure 2C**).

**Figure 3:**
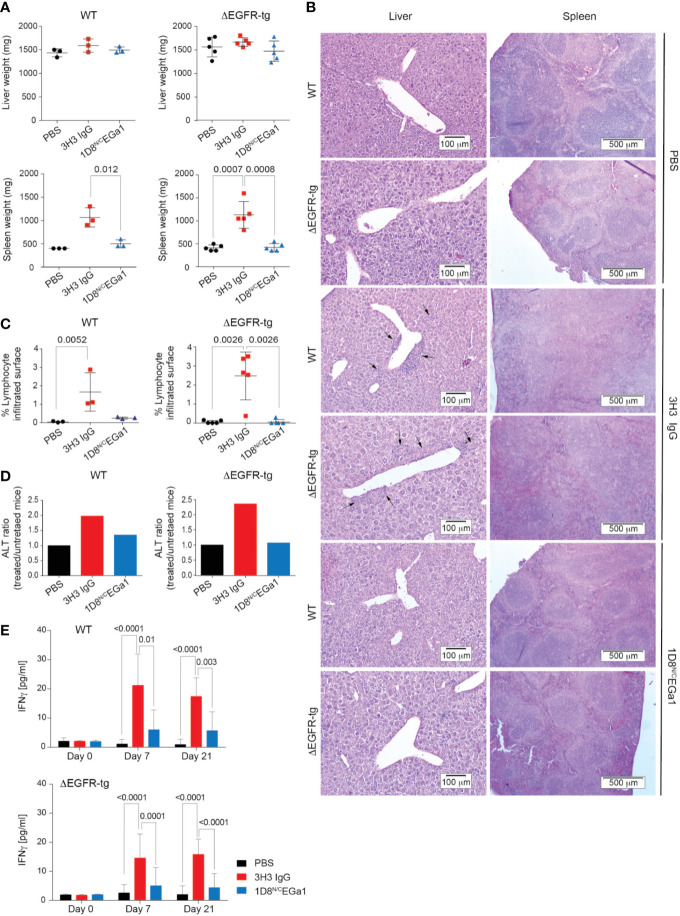
Treatment with 1D8^N/C^EGa1 does not induce toxicity. **(A)** Liver and spleen weights from WT and ΔEGFR-tg mice littermates (*n* = 3–5/group) treated with PBS, 3H3 IgG, or 1D8^N/C^EGa1. **(B)** Hematoxylin and eosin staining of representative tissue slides from the liver, and spleen of mice treated with PBS, 3H3 IgG, and 1D8^N/C^EGa1. Scale bars are shown. **(C)** Quantification of the mononuclear cell infiltrated surface in the liver of WT (*n* = 3 per treatment) and ΔEGFR-tg (*n* = 5 per treatment) littermates treated with PBS, 3H3 IgG, or 1D8^N/C^EGa1. **(D)** Serum ALT levels in response to the treatments with 3H3 IgG and 1D8^N/C^EGa1. The ratio of ALT activity of WT and ΔEGFR-tg mice treated with 3H3 IgG or 1D8^N/C^EGa1 *vs.* the ALT activity of WT and ΔEGFR-tg mice treated with PBS at day 14 of treatments is shown (*n* = 3/5 per treatment). **(E)** Sera from WT and ΔEGFR-tg mice were collected from peripheral blood at days 0, 7, and 21 of treatment, and levels of IFNγ were measured by ELISA (*n* = 3 per time point). Results represent 2 separate experiments. All data are presented as mean ± SD. *P* values were calculated with Student’s *t* test.

We compared the toxicity profile of the 1D8^N/C^EGa1 trimerbody with that of the well-characterized anti-4-1BB agonistic 3H3 IgG (4) in WT and ΔEGFR mice injected (6 mg/kg) i.p. once a week for 3 weeks and euthanized 1 week later. As shown in **Figure 3A**, treatment of ΔEGFR-tg mice with 3H3 IgG resulted in significant enlargement of the spleen as demonstrated by weight (*P* = 0.0008). In contrast, treatment with 1D8^N/C^EGa1 did not result in splenomegaly or hepatomegaly (**Figure 3A**). The histologic study of the liver of mice treated with 3H3 IgG revealed significant mononuclear cell infiltration, forming periportal cuffs with thickening of tunica media and also infiltration foci associated with microvasculature, while no significant infiltration was observed in mice treated with 1D8^N/C^EGa1 (**Figure 3B**). Indeed, the surface of infiltrating mononuclear cells accounted for around 2.5% of the liver of ΔEGFR-tg mice treated with 3H3 IgG, while it only represented 0.06% in mice treated with 1D8^N/C^EGa1 (*P* = 0.0026) or PBS (*P* = 0.0026) (**Figure 3C**). Consistent with these results, we observed a 2-fold increase in alanine transaminase (ALT) levels in the serum of WT and ΔEGFR-tg mice treated with 3H3 IgG compared to mice of the same genotype treated with PBS. In contrast, mice treated with 1D8^N/C^EGa1 showed little or no increase in ALT levels (**Figure 3D**). The effect of treatment with 3H3 IgG or 1D8^N/C^EGa1 on the levels of IFNγ in serum was also compared. 3H3 IgG treatment triggered significant elevation of IFNγ at days 7 and 21 in both WT and ΔEGFR-tg mice (**Figure 3E**). In contrast, 1D8^N/C^EGa1 induced levels of IFNγ comparable to PBS-treated mice in both groups.

In summary, we demonstrated that treatment of ΔEGFR-tg mice with the strong 4-1BB-agonistic 3H3 IgG induced a toxicity profile similar to that observed in WT C57BL/6 mice, with significant immune cell infiltration and systemic inflammation, indicating the suitability of the model to study 4-1BB–related toxicity. In contrast, none of these features were observed in ΔEGFR-tg mice treated with the 1D8^N/C^EGa1 trimerbody, despite the expression of both huEGFR and moEGFR on the hepatocyte surface, which excludes that the lower affinity of 1D8^N/C^EGa1 for moEGFR may be responsible for the absence of liver toxicity observed in WT mice. These results further support the role of FcγR interactions in the 4-1BB-agonist-associated immunological abnormalities and organ toxicities (20–22) and confirm the safety profile of EGFR-targeted 4-1BB-agonistic trimerbodies in systemic cancer immunotherapy protocols.

## Data Availability Statement

The raw data supporting the conclusions of this article will be made available by the authors, without undue reservation.

## Ethics Statement

The animal study was reviewed and approved by the Animal Experimentation Ethics Committee of the Instituto de Investigaciones Biomédicas Alberto Sols, and the Animal Welfare Division of the Environmental Affairs Council of the Government of Madrid (66/14, 118/19).

## Author Contributions

MC, JMZ, and LA-V designed and supervised the study. MC, SLH, JM-T, GP-C, PG-G, and AT-G performed the core experiments. MC, GP-C, and JMZ were responsible for the animal experiments. PG-G and JM-T performed IHC analysis. MC, SLH, JM-T, PMPVBEH, AS, IF, LS, JMZ, and LA-V provided scientific feedback, discussed the data, and wrote the manuscript. All authors contributed to the article and approved the submitted version.

## Funding

This study was supported by grants from the European Union [IACT Project (602262)], the Spanish Ministry of Science and Innovation; the Spanish Ministry of Economy and Competitiveness (SAF2017-89437-P, PID2019-110405RB-100, RTC-2016-5118-1, RTC-2017-5944-1), partially supported by the European Regional Development Fund; the Carlos III Health Institute (PI16/00357), co-founded by the Plan Nacional de Investigación and the European Union; the CRIS Cancer Foundation (FCRIS-IFI-2018), and the Spanish Association Against Cancer (AECC, 19084).

## Conflict of Interest

MC is an employee of Leadartis. LA-V and LS are co-founders of Leadartis.

The remaining authors declare that the research was conducted in the absence of any commercial or financial relationships that could be construed as a potential conflict of interest.
