# Supplementary material for: Case Report: An EGFR-Targeted 4-1BB-agonistic Trimerbody Does Not Induce Hepatotoxicity in Transgenic Mice With Liver Expression of Human EGFR
Source: Front Immunol. 2021 Jan 7;11:614363. doi: 10.3389/fimmu.2020.614363 (PMC7817978; doi:10.3389/fimmu.2020.614363)
Supplement: Supplementary file 1 [file DataSheet_1.pdf]

Supplementary Material

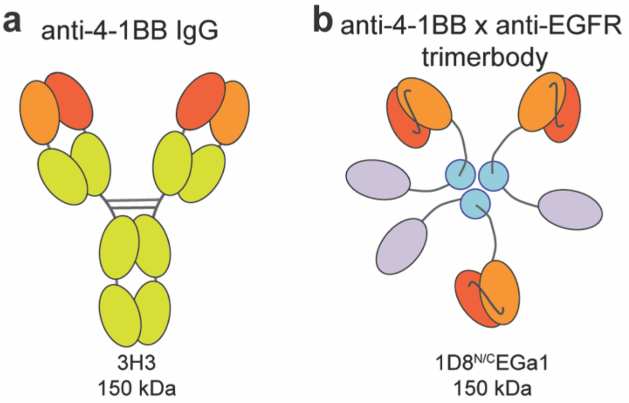

Supplementary Figure 1. Schematic diagrams showing the protein structure of the anti-4-1BB 3H3 IgG (a), and the anti-4-1BB x anti-EGFR bispecific 1D8$^{N/C}$EGa1 trimerbody (b). The variable regions derived from anti-4-1BB antibodies are represented in orange, the anti-EGFR V_{HH} EGa1 in magenta. The structural domains of the IgG are represented in light-green and the homotrimerization domains of the trimerbody in light-blue. The linker regions are in gray.

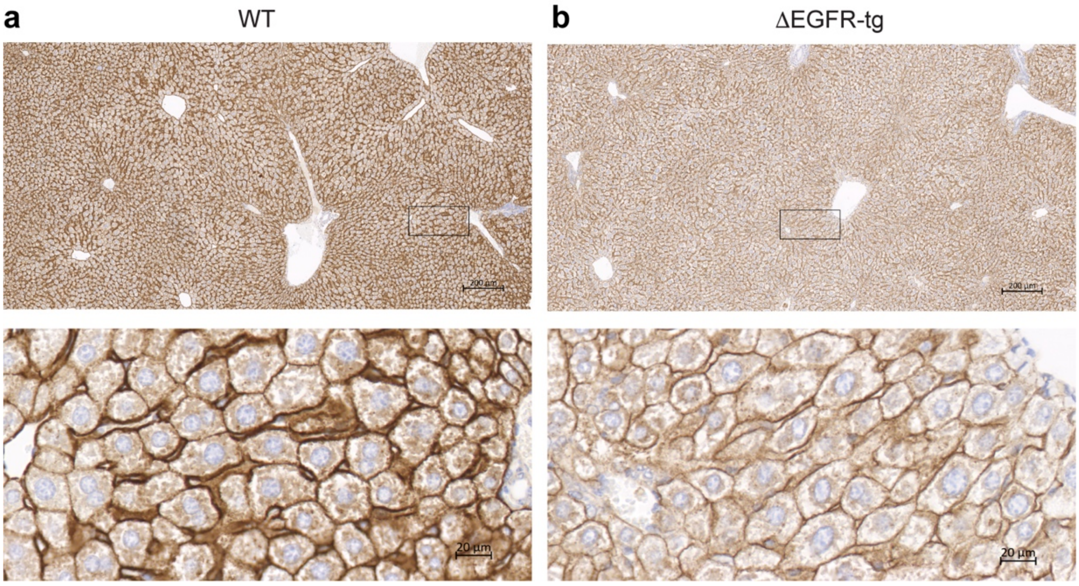

Supplementary Figure 2. Analysis of mouse EGFR expression by IHC in liver sections from (**a**) wild-type C57BL/6 (WT) and (**b**) ΔEGFR-tg mice. Representative micrographs of liver sections from the indicated mice showing mouse EGFR staining in brown. Nuclei are counterstained with hematoxylin. Scale bars, 200 µm (upper images); and 20 µm (lower images). Upper panels show panoramic views of the corresponding mouse livers, and lower panels are higher magnification images corresponding to zones indicated by black boxes.
